# Evaluating the consistency of gene sets used in the analysis of bacterial gene expression data

**DOI:** 10.1186/1471-2105-13-193

**Published:** 2012-08-08

**Authors:** Nathan L Tintle, Alexandra Sitarik, Benjamin Boerema, Kylie Young, Aaron A Best, Matthew DeJongh

**Affiliations:** 1 Department of Mathematics, Statistics and Computer Science, Dordt College, Sioux Center, IA, 51250, USA; 2 Department of Biostatistics, University of Michigan, Ann Arbor, MI, 43109, USA; 3 Department of Industrial and Operations Engineering, University of Michigan, Ann Arbor, MI, 48109, USA; 4 Department of Mathematics, Hope College, Holland, MI, 49423, USA; 5 Department of Biology, Hope College, Holland, MI, 49423, USA; 6 Department of Computer Science, Hope College, Holland, MI, 49423, USA

**Keywords:** Gene ontology, KEGG, SEED, Operons, Consistency

## Abstract

**Background:**

Statistical analyses of whole genome expression data require functional information about genes in order to yield meaningful biological conclusions. The Gene Ontology (GO) and Kyoto Encyclopedia of Genes and Genomes (KEGG) are common sources of functionally grouped gene sets. For bacteria, the SEED and MicrobesOnline provide alternative, complementary sources of gene sets. To date, no comprehensive evaluation of the data obtained from these resources has been performed.

**Results:**

We define a series of gene set consistency metrics directly related to the most common classes of statistical analyses for gene expression data, and then perform a comprehensive analysis of 3581 Affymetrix® gene expression arrays across 17 diverse bacteria. We find that gene sets obtained from GO and KEGG demonstrate lower consistency than those obtained from the SEED and MicrobesOnline, regardless of gene set size.

**Conclusions:**

Despite the widespread use of GO and KEGG gene sets in bacterial gene expression data analysis, the SEED and MicrobesOnline provide more consistent sets for a wide variety of statistical analyses. Increased use of the SEED and MicrobesOnline gene sets in the analysis of bacterial gene expression data may improve statistical power and utility of expression data.

## Background

Within the last decade, microarrays measuring whole genome transcript abundance (gene expression arrays) have grown in popularity. Increasingly, the data received from this maturing technology is of high quality. However, these data are only useful if they are interpretable biologically. In order to draw biologically relevant conclusions from gene expression data, a variety of statistical analysis methods can be used.

Traditional methods for the statistical analysis of bacterial gene expression data include creating rank-ordered lists of differentially expressed genes (log-ratio of genes in two experiments), *k*-means clustering [1], principal components analysis [2], on/off calling algorithms used in flux-balance analysis [e.g., [3,4] and regulatory network inference (RNI) [e.g., [5,6]. Newer methods include gene set or pathway analysis [e.g., [7-9], alternative clustering methods [e.g., [10] and integrated regulatory/metabolic modeling approaches [e.g., [11,12].

Regardless of how bacterial gene expression data is analyzed, high-quality biological information (e.g., gene function) is critical to the ultimate utility of gene expression data. Increasingly, the Gene Ontology [GO; [13] and Kyoto Encyclopedia of Genes and Genomes [KEGG; [14] are used as a primary source of biological information for a variety of gene expression data analysis approaches. However, to date and despite their popularity, little effort has been put into evaluating the consistency of gene sets from GO or KEGG for bacterial organisms as compared to other options (e.g., SEED based sets [15] and MicrobesOnline [16] operon predictions).

In this paper we define a variety of gene set consistency metrics that are directly related to three classes of statistical analysis methods. These metrics enable us to measure the degree to which a given gene set is likely to be informative in the context of the respective analyses. In short, more consistent gene sets will behave in statistically optimal ways. In this manuscript we use 3581 Affymetrix® whole genome expression arrays for 17 different bacteria to evaluate the consistency of gene sets from GO, KEGG, SEED and MicrobesOnline. We find that certain sources of bacterial gene sets yield highly consistent sets across all metrics.

## Methods

### Gene expression data

Seventeen diverse bacteria were selected for inclusion in this study based on the availability of Affymetrix GeneChip® cell intensity (CEL) files for substantial numbers of microarray experiments. We obtained these CEL files from multiple sources: NCBI’s Gene Expression Omnibus [GEO; [17], the Many Microbe Microarrays Database [M3D; [18], and, in one case, a private laboratory (Dr. Paul Dunman, personal communication). Expression data from GEO and M3D is available via direct download from those repositories, while expression data from the private laboratory is available from Dr. Paul Dunman upon request. Table 1 lists the 17 organisms included in this study, the number of CEL files available for each organism (# arrays), and the source of the CEL files for each organism. Minimal strain and platform differences are present within each set of CEL files for a given organism; however, any differences were ignored for our analysis. For each organism, the expression data from the CEL files was background corrected, normalized and summarized using Robust Multichip Averaging [19] as implemented in R/Bioconductor [20] using the rma() function default settings. In addition, the probe sets for each Affymetrix GeneChip were mapped to gene identifiers in the SEED [15] in order to provide a consistent basis for analyzing each source of gene sets. The number of genes measured by the CEL files is also included in the table. The final analysis dataset for each organism consists of a two-dimensional table containing a single expression value for each SEED gene identifier (row) and CEL file (column).

**Table 1:** Characteristics of the set of microarrays

**Organism name**	**# of genes**^**1**^	**# of arrays**	**Source**^**2**^	**Number of Gene sets**								
				**Gene Ontology**			**KEGG maps**^**3**^	**MO: Predicted Operons**^**3**^	**SEED**^**3**^			**Total**
				**BP**^**3**^	**CC**^**3**^	**MF**^**3**^			**SS**^**3**^	**Scenario**^**3**^	**Path**^**3**^	
**Actinobacteria**												
*Streptomyces coelicor* A3(2)	7989	55	GEO	799	117	819	113	1626	200	139	158	3971
**Bacteroidetes**												
*Bacteroidesthetaiotaomicron* VPI-5482	4778	41	GEO	695	84	615	79	1008	193	93	98	2865
**Cyanobacteria**												
*Synechococcuselongatus* PCC 7942	1849	104	GEO	651	106	564	89	295	257	72	90	2124
**Deinococcus-Thermus**												
*Thermusthermophilus*	2215	407	GEO	604	79	499	83	441	180	86	92	2064
**Firmicutes**												
*Staphylococcus aureus*subsp. aureus Mu50	2750	852	PD	697	86	604	90	521	360	98	118	2574
*Streptococcus agalactiae*	1880	78	GEO	571	65	477	79	257	238	64	70	1830
*Streptococcus pyogenes*	1849	89	GEO	556	70	488	81	248	256	57	73	1829
**Mollicutes**												
*Mycoplasma pneumoniae* M129	731	43	GEO	332	46	252	42	80	80	14	14	860
**Proteobacteria**												
**Alpha**												
*Bradyrhizobiumjaponicum*	8198	195	GEO	457	111	547	103	1672	421	138	156	3605
*Rhodobactersphaeroides*	4084	119	GEO	767	150	724	103	757	367	110	128	3106
*Rickettsia rickettsii*str.Iowa	1242	100	GEO	445	64	367	59	251	135	14	15	1350
**Epsilon**												
*Helicobacter pylori* HPAG1	1521	56	GEO	572	76	426	77	248	174	42	42	1657
**Gamma**												
*Escherichia coli* K12	4329	907	M3D	847	115	788	98	752	399	160	169	3328
*Pasteurella multocida* subsp. multocida str. Pm70	2001	72	GEO	653	77	673	81	365	328	88	91	2356
*Pseudomonas aeruginosa* PA01	5598	176	GEO	823	133	835	104	1042	389	136	154	3616
*Shewanellaoneidensis* MR-1	4050	245	M3D	746	112	641	95	705	328	109	125	2861
*Vibrio parahaemolyticus* RIMD 2210633	4515	42	GEO	719	109	602	93	920	418	144	165	3170
**Total**	**--**	3581	**--**	10934	1600	9921	1469	11188	4723	1564	1767	43166

^1^ For which array data is available.

^2^*M3D*=Many Microbe Microarrays Database [18], *PD*=The Paul Dunman Laboratory, *GEO*=Gene Expression Omnibus [17].

^3^ Gene Ontology [13], *BP*=Biological Process, *CC*=Cellular Component, *MF*=Molecular Function, *KEGG*=Kyoto Encyclopedia of Genes and Genomes [14], *MO*= Microbes Online Predicted Operons using the method of Price et al. [16,21], SEED [15,22], *SS*=SEED subsystems, Scenario=SEED scenario, *Path*=SEED path.

### Gene sets

For each organism, we obtained gene sets using four different resources: GO [13], KEGG [14], MicrobesOnline [16], and the SEED [15]. We obtained GO annotations from MicrobesOnline, and mapped KEGG and MicrobesOnline gene identifiers to SEED gene identifiers using locus tags. GO defines hierarchies of terms for molecular function (MF), biological process (BP), and cellular component (CC), arranged in three separate directed acyclic graphs. We created a gene set for each GO term by gathering all of the organism’s genes associated with the given GO term or with any of the GO term’s children. KEGG defines metabolic pathway maps representing networks of biochemical reactions catalyzed by the enzymes encoded in the organism’s genome. We created gene sets from each map by gathering all genes associated with reactions in that map. MicrobesOnline (MO) uses the method of Price et al. [21] to create MO Predicted Operons; we created a gene set for each Predicted Operon for the organism. The SEED organizes functional roles for genes into Subsystems (SS) that represent components of cellular processes and cellular structures; we created a gene set for each subsystem for a given organism by gathering all genes annotated with functional roles represented in that subsystem. SEED subsystems that represent metabolic processes are further subdivided into Scenarios, which define input and output compounds along with the subsets of functional roles that are associated with particular components of the metabolic process. Scenarios can be subdivided further into one or more Paths, which define the alternative minimal subsets of functional roles that connect scenario input and output compounds (see example below) [22]. We created gene sets for each Scenario and for each Path by gathering all genes annotated with the respective functional roles. In total, across the 17 organisms, we gathered 43,166 distinct gene sets containing at least two genes with gene expression data available (see Table 1).

### Example of gene sets for arginine biosynthesis

Consider the gene that encodes argininosuccinate synthase in *Escherichia coli str. K-12 substr. MG1655*. The SEED identifier for this gene is fig|83333.1.peg.3116, and the MicrobesOnline identifier is VIMSS17244. According to MicrobesOnline, this gene is annotated with three GO terms: *GO:0004055 argininosuccinate synthase activity (MF)*, *GO:0006526 arginine biosynthetic process (BP),* and *GO:0005524 ATP binding (MF)*. Each of these three GO terms is associated with multiple genes in *E. coli K-12*: (1) the gene set for *GO:0004055 argininosuccinate synthase activity* is composed of three genes; (2) the gene set for *GO:0006526 arginine biosynthetic process* also contains these three genes, as well as six other genes in this metabolic pathway; and (3) term *GO:0005524 ATP binding* represents a very broad set of 336 genes. In addition, each of these GO terms has parent terms in their respective GO hierarchies, and the gene sets for the parent terms form supersets of the gene sets described here. For example, one of the parent terms for *GO:0006526 arginine biosynthetic process* is *GO:0006525 arginine metabolic process (BP)*, which contains genes associated with both arginine biosynthesis as well as arginine degradation. In all, the gene *fig|83333.1.peg.3116* is present in 41 gene sets derived from the GO.

The gene is associated with two KEGG maps, both of which represent areas of metabolism that extend well beyond arginine biosynthesis, so their respective gene sets for *E. coli K-12* are correspondingly large: *Alanine, aspartate and glutamate metabolism* (29 genes) and *Arginine and proline metabolism* (43 genes).

This gene is a member of one subsystem in the SEED, *Arginine Biosynthesis extended,* which contains the eleven genes shown in Table 2. Because this subsystem contains functional roles corresponding to the biological process of arginine biosynthesis, the derived gene set is similar to the gene set for the GO term *GO:0006526 arginine biosynthetic process* though not exactly the same (see Table 2). In particular, note that four of the eleven genes are missing from the *GO:0006526 arginine biosynthetic process* gene set, even though three of them encode enzymes that are necessary components of the arginine biosynthesis process. Similarly, the KEGG maps either miss vital genes (e.g., *eco00250*) or include many other genes (35 additional genes in *eco00330*).

**Table 2:** Example of overlap among gene sets related to arginine biosynthesis

**GeneID from the SEED**	**Functional role**	**Arginine Biosynthesis Extended (SEED: SUBS)**	**GO: 0006526(Arginine Biosynthetic Process; BP)**	**Alanine, aspartate and glutamate metabolism (eco00250: KEGG)**	**Arginine and Proline Metabolism (eco00330: KEGG)**	**Glutamate to Arginine (SEED: Scenario, Path)**	**MO: Predicted Operons**
fig|83333.1.peg.269	Ornithine carbamoyltransferase (EC 2.1.3.3)	+	M	M	+	+	None
fig|83333.1.peg.2440	N-succinyl-L,L-diaminopimelatedesuccinylase (EC 3.5.1.18)	+	+	M	M	M	with peg.2339
fig|83333.1.peg.2771	N-acetylglutamate synthase (EC 2.3.1.1)	+	+	M	+	+	None
fig|83333.1.peg.3116	Argininosuccinate synthase (EC 6.3.4.5)	+	+	+	+	+	None
fig|83333.1.peg.3181	Arginine pathway regulatory protein ArgR, repressor of argregulon	+	M	M	M	M	None
fig|83333.1.peg.3294	Acetylornithine and N-succinyl-L,L-diaminopimelateaminotransferase (EC 2.6.1.11 and EC 2.6.1.17)	+	M	M	+	+	None
fig|83333.1.peg.3877	Acetylornithinedeacetylase (EC 3.5.1.16)	+	+	M	+	+	None
fig|83333.1.peg.3878	N-acetyl-gamma-glutamyl-phosphate reductase (EC 1.2.1.38)	+	+	M	+	+	with peg.3879 and peg.3880
fig|83333.1.peg.3879	Acetylglutamate kinase (EC 2.7.2.8)	+	+	M	+	+	with peg.3878 and peg.3880
fig|83333.1.peg.3880	Argininosuccinatelyase (EC 4.3.2.1)	+	+	+	+	+	with peg.3878 and peg.3879
fig|83333.1.peg.4164	Ornithine carbamoyltransferase (EC 2.1.3.3)	+	M	M	+	+	None
	Number of other genes in the set^1^	0	2	28	34	0	

^1^Genes in the set that don’t appear in this table.

+ means present in the set.

M means Missing (not present in the set).

None means that the gene is not present in any predicted operon.

There is one scenario in the *Arginine Biosynthesis extended* subsystem, named *Glutamate to Arginine*; it defines the functional roles in the subsystem corresponding to enzymes that are specifically involved in synthesizing L-arginine from L-glutamate. The gene set derived from this scenario is a proper subset of the gene set derived from the subsystem.

Although there are two possible Paths through the *Glutamate to Arginine* scenario, one using Acetylornithine deacetylase (EC 3.5.1.16) and the other using Glutamate N-acetyltransferase (EC 2.3.1.35) to convert N-acetylornithine to L-ornithine, *E coli K-12* only encodes one of these enzymes; thus the gene set for the Path is exactly the same as the gene set for the scenario itself.

Lastly, Table 2 illustrates which of the genes involved in arginine biosynthesis are predicted by MicrobesOnline to be in an operon. There is one complete operon in the table (fig|83333.1.peg.3878, fig|83333.1.peg.3879 and fig|83333.1.peg.3880) and one partial operon (fig|83333.1.peg.2440 is in an operon with fig|83333.1.peg.2339). We note that fig|83333.1.peg.3877 is on the opposite strand from fig|83333.1.peg.3878, fig|83333.1.peg.3879 and fig|83333.1.peg.3880 and, thus, cannot be in the same operon.

### Consistency metrics for gene expression data analysis

We developed four different classes of metrics to measure the consistency of gene sets in the context of common types of statistical analyses: metrics for differential expression, absolute expression and correlation (either magnitude only, or magnitude and consistency). In the following sections, we describe the four classes of metrics and how each reflects the characteristics and assumptions of a statistical method.

### Differential expression values

In a differential expression analysis, a researcher creates a rank-ordered list of differential expression values *D = (d*_*1*_*,…,d*_*M*_*),* comparing two experiments *k* and *l*, where *M* is the number of genes for which expression data is available, and di=ek,iel,i, where *e*_*k,i*_ is the normalized, background corrected expression value for the *i*^*th*^ gene in experiment *k*.

A generic statistical model for the observed differential expression for all genes *ii* = 1,…,*n,* in the set of interest, *N*, which contains *n* genes, is $d_{i}=\alpha +{\left(\beta +\epsilon \right)}_{i}$, where α equals the overall differential expression effect for the set of *n* genes, *β* is the additional differential expression effect for gene *i*, and *ϵ* is a random error term for gene *i*. In order to maximize statistical power to estimate *α,*(*β+ϵ*) should be minimized [e.g., [7]. Thus, we propose measuring the consistency of gene expression data as a way to assess statistical power of different sources of sets used in a differential expression analysis.

We propose two different metrics to assess the spread of differential expression values: *s*_*mean,diff*_ and *s*_*median,diff*_. To obtain these values, we first compute the standard deviation of differential expression values across the genes in a set of interest. Specifically, we find the standard deviation of {*d*_*i*_*,.d*_*n*_} *= s*_*N,(k,l)*_ for the gene set of interest, *N*, for a pair of experiments (*k, l*). We then find the average (*s*_*mean,diff*_) and median (*s*_*median,diff*_) of the standard deviations across pairs of experiments. Specifically we define $s_{mean,diff}=\frac{\sum_{k=1}^{P-1}\sum_{l=k+1}^{P}s_{N,\left(k,l\right)}}{P\left(P-1\right)/2}$, where *P* = the total number of arrays and *s*_*median,diff*_ as the median of *s*_*N,(K,L)*_ across all unique pairs of experiments *(k,l)*. Smaller values of *s*_*mean,diff*_ and *s*_*median,diff*_ indicate more consistent gene sets. We used random sampling to generate unbiased estimates of *s*_*median,diff*_ and *s*_*median,diff*_ for all for 43,166 gene sets, by randomly selecting 100 pairs of microarrays for each organism.

### Absolute expression values

In some cases, for example when estimating when a gene is on or off in flux-balance analysis [e.g., [3,4], researchers look directly at the rank ordered list of expression values *E* = (*e*_*k,1, ,*_*e*_*kj,m*_*)* to determine whether genes have high or low expression levels. Gene set analysis can also be applied to *E*[7]. We can statistically model the expression values as $e_{i}=\alpha +{\left(\beta +\epsilon \right)}_{i}$ for the *i*^*th*^ of *n* genes in the set of interest. Using similar rationale to the previous section, statistically optimal sets of genes will show high consistency in values of *e*_*i*_.

We propose two measures of gene set consistency related to spread of absolute expression data. For each set of genes, we computed the mean (*s*_*mean,exp*_) and median (*s*_*median,exp*_) standard deviations of the expression values for all genes in a set across all arrays for an organism. Specifically, we first find the standard deviation of the expression values for all *n* genes in a set of interest, *N*, for each array *k* {*e*_*i*_*,.e*_*n*_} *= s*_*N,k*_. These standard deviations are then either averaged, across the *P* arrays, or the median of *s*_*N,K*_ (*s*_*median,exp*_) across the *P* arrays is computed. Smaller values of these two measures will be obtained for sets with more consistent levels of expression. We computed both metrics for all gene sets in our analysis.

### Correlation between expression values

K-means and other clustering algorithms [e.g., [1,2], operon prediction algorithms [e.g., [21] and regulatory network inference [e.g., [5,6], typically require a dataset of correlations between the expression values of pairs of genes. In general, these methods operate on a rank ordered list of pairwise gene correlations (e.g., Pearson) and attempt to find pairs of genes showing strong pairwise correlations in order to argue that the pair of genes is co-regulated. Similar to the previous two sections, we can model the observed correlation *ρ*_*i,j*_ between a pair of genes *i* and *j* which are both members of a gene set of interest, as $\rho_{i,j}=\alpha +{\left(\beta +\epsilon \right)}_{i,j}$. Methods that use pairwise gene correlations operate under the assumption that sets of genes demonstrating high and consistent average pairwise correlations are biologically meaningful.

### Correlation between expression values: magnitude only

In order to measure gene set consistency based on correlation of expression values, we computed the average pairwise Pearson correlation (*corr*_*mean*_) and median pairwise Pearson correlation (*corr*_*median*_) across all pairs of genes in the set of interest across all experiments. Specifically, $corr_{\mathrm{mean}}=\frac{\sum_{j=1}^{n-1}\sum_{i=j+1}^{n}\left|r_{i,j}\right|}{n\left(n-1\right)/2}$, where *r*_*i,j*_ is the Pearson correlation between genes *i* and *j* for the available set of expression arrays, and *n* = the number of genes in the set of interest. *corr*_*median*_ is the median value of *r*_*i,j*_ where *r*_*i,j*_ is computed for each unique pair of *i* and *j*, across the set of *n* genes in the set. Highly consistent gene sets will yield larger values of *corr*_*mean*_ and *corr*_*median.*_ For all sets of more than 50 genes, we utilized random sampling to generate unbiased estimates of *corr*_*mean*_ and *corr*_*median*_ by selecting a random subset of 50 genes.

### Correlation between expression values: magnitude and consistency

We computed an additional measure of gene set consistency that recognizes that high average pairwise correlation are optimal, but that optimal gene sets will also show consistently high correlation based on principal components analysis (PCA). PCA was applied to the variance-covariance matrix of gene pairs within the gene set of interest *N*, across the entire set of arrays *P* available for the organism using the *prcomp* function in R [23]. PCA attempts to explain the variance-covariance matrix (correlation structure) of a set of variables (in this case variables are genes) through as few linear combinations of the variables as possible. The metric we used to summarize gene set consistency is the percent of variation explained by the first principal component (linear combination), *PC*_*1*_. Larger values of *PC*_*1*_ indicate stronger and more consistent correlation between all of the genes in the set.

### Using the consistency metrics

In our analyses, we computed the values of the consistency metrics for each of the 43,166 gene sets across the set of available expression data. We then explored patterns in the values of the consistency metrics to assess whether certain sources of gene sets provided more consistent sets of genes.

## Results

### Sample characteristics

As shown in Table 1, there were 43,166 sets of at least two genes across the eight different types of gene sets. The number of sets from each source ranged from 1,469 sets (KEGG) to 11,188 sets (MicrobesOnline Predicted Operons). The number of sets for each organism is strongly related to the number of genes in the organism (*r* = 0.92, *p* < 0.001).

Set sizes ranged from 2 to 3,661 genes. Table 3 shows how set sizes differed by organism and by source. In general, Predicted Operons, Scenarios and Paths were smaller than subsystems and sets based on GO and KEGG. While some differences in set size were observed by organism (corresponding to the number of genes in the organism), two separate regression models predicting set size (log_10_ scaled) by either organism type or source of sets, indicate that the source of sets accounts for substantially more difference in set size, than does the organism (organism type: *p* = 4.1x10^-15^; *r*^*2*^ = 0.002; source of sets: *p* < 2.2x10^-16^, *r*^*2*^ = 0.13).

**Table 3:** **Median (and maximum**^**1**^**) of set sizes by organism and source**

	**Gene ontology**			**KEGG**^**2**^	**MO: Predicted Operons**^**2**^	**SEED**		
	**BP**^**2**^	**CC**^**2**^	**MF**^**2**^			**SS**^**2**^	**Scen**^**2**^	**Path**^**2**^
**Actinobacteria**								
*Streptomyces coelicor* A3(2)	8 (3661)	5 (1574)	5 (4487)	15 (192)	3 (42)	7 (57)	5 (28)	5 (28)
**Bacteroidetes**								
*Bacteroidesthetaiotaomicron* VPI-5482	7 (2141)	7.5 (1037)	5 (2580)	13 (53)	3 (32)	5 (74)	4 (17)	4 (17)
**Cyanobacteria**								
*Synechococcuselongatus* PCC 7942	7 (1268)	11 (580)	4 (1498)	10 (66)	3 (18)	6 (47)	4 (13)	5 (13)
**Deinococcus-Thermus**								
*Thermusthermophilus*	6 (1039)	6 (468)	4 (1242)	10 (63)	2 (32)	5 (39)	3 (15)	3 (15)
**Firmicutes**								
*Staphylococcus aureus*subsp. aureus Mu50	8 (1413)	4.5 (711)	5 (1596)	11 (107)	2 (23)	5 (35)	4 (12)	4 (12)
*Streptococcus agalactiae*	6 (1064)	16 (554)	5 (1229)	8 (97)	4 (53)	5 (32)	3 (10)	4 (10)
*Streptococcus pyogenes*	6 (965)	11.5 (503)	5 (1138)	9 (66)	4 (46)	5 (34)	4 (10)	4(10)
**Mollicutes**								
*Mycoplasma pneumoniae* M129	5 (350)	9 (196)	5 (407)	5 (52)	5 (66)	4 (32)	4.5 (9)	4.5 (9)
**Proteobacteria**								
**Alpha**								
*Bradyrhizobiumjaponicum*	7 (1578)	3 (703)	5 (2184)	24 (354)	3 (36)	8 (66)	4 (37)	4 (37)
*Rhodobactersphaeroides*	7 (2076)	4 (1054)	4 (2480)	15 (202)	3 (52)	6 (58)	3 (14)	3 (14)
*Rickettsia rickettsii*str.Iowa	7 (549)	10 (315)	4 (622)	6 (53)	3 (29)	4 (32)	3.5 (13)	4 (13)
**Epsilon**								
*Helicobacter pylori* HPAG1	6 (815)	11.5 (418)	4.5 (932)	10 (52)	4 (29)	5 (37)	3 (9)	3 (9)
**Gamma**								
*Escherichia coli* K12	7 (2370)	4 (1308)	4 (2665)	14 (180)	2 (28)	6 (63)	3.5 (18)	3 (18)
*Pasteurella multocida* subsp. multocida str. Pm70	7 (1207)	10 (690)	4 (1412)	11 (124)	4 (33)	5 (33)	3 (12)	3 (12)
*Pseudomonas aeruginosa* PA01	8 (2981)	4 (1626)	4 (3511)	17 (200)	2 (31)	7 (58)	4 (26)	4 (26)
*Shewanellaoneidensis* MR-1	7 (1959)	5.5 (1024)	5 (2237)	14 (105)	2 (28)	7 (60)	4 (15)	4 (14)
*Vibrio parahaemolyticus* RIMD 2210633	7 (1618)	5 (896)	4 (1771)	12 (87)	3 (46)	6 (83)	4 (15)	4 (15)
**Total**	7 (3661)	6.5 (1626)	4 (4487)	12 (354)	3 (66)	6 (83)	4 (37)	4 (37)

^1^ The minimum set size in all cases was 2.

^2^Gene Ontology [13], *BP*=Biological Process, *CC*=Cellular Component, *MF*=Molecular Function, *KEGG*=Kyoto Encyclopedia of Genes and Genomes [14], *MO*= Microbes Online Predicted Operons using the method of Price et al. [16,21], SEED [15,22], *SS*=SEED subsystems, Scenario=SEED scenario, *Path*=SEED path.

### Characteristics of the consistency metrics

Table 4 shows how the values of the seven different consistency metrics correlated with each other across all gene sets considered in this analysis. Within each of the three general types of approaches for computing consistency (Differential Expression, Absolute Expression and Correlation) the correlation between consistency metrics is quite high. In particular, mean and median metrics are so strongly correlated that, in general, we can conclude that skewness/outliers are not significantly impacting the consistency metrics. Thus, we will focus our analysis only on the three mean based metrics and *PC*_*1*_ from this point forward in the manuscript. Between the three classes, the consistency metrics show only weak to moderate correlation. This suggests that while there is some overall notion of consistent gene sets regardless of the metric used, there are still many sets that will appear to be consistent using one metric and not consistent using another metric. For example, a set with high correlation (high *corr*_*mean*_)but low consistency in absolute expression (high *s*_*mean,exp*_) could occur if the genes in the set are co-regulated, but some genes in the set typically show high expression levels, while other genes in the set typically show low expression levels. We also note that the negative correlation between differential/absolute expression consistency metrics and correlation consistency metrics is expected because differential/absolute expression consistency metrics take small values when applied to consistent gene sets, while correlation consistency metrics take large values when applied to consistent gene sets.

**Table 4:** Pearson correlations between consistency metrics

	**Differential expression**		**Absolute expression**		**Correlation**		
					**Magnitude only**		**Magnitude and consistency**
	***s***_***mean,diff***_	***s***_***median,diff***_	***s***_***mean,exp***_	***s***_***median,exp***_	***corr***_***mean***_	***corr***_***median***_	***PC***_*1*_
*s*_*mean,diff*_	1.0						
*s*_*median,diff*_	0.95	1.0					
*s*_*mean,exp*_	0.38	0.40	1.0				
*s*_*median,exp*_	0.36	0.39	0.99	1.0			
*corr*_*mean*_	-0.15	-0.21	-0.30	-0.28	1.0		
*corr*_*median*_	-0.15	-0.21	-0.29	-0.28	0.98	1.0	
*PC*_*1*_	-0.30	-0.42	-0.43	-0.43	0.64	0.62	1.0

### Consistency of expression level by source

Using the four consistency metrics to which we are restricting the remainder of our analyses, Table 5 illustrates the consistency of gene sets from each of the eight set sources. Specifically, Table 5 gives the mean of the consistency metrics across all sets and organisms within each of the eight set sources. Importantly, results are similar for each of the four different consistency metrics. Namely, Predicted Operons are the most consistent, followed by Paths and Scenarios. Subsystems tended to perform slightly better than GO sets, and KEGG was typically worst. Cellular component sets performed similar to SEED sets when using correlation based consistency metrics. Results using the three median based consistency metrics yielded similar patterns (results not shown).

**Table 5:** Mean levels of consistency metrics by source (rank out of the 8 sources in parentheses)

		***s***_***mean,diff***_^**a**^	***s***_***mean,exp***_^**a**^	***corr***_***mean***_^***b***^	**PC**_**1**_^**b**^
Gene Ontology	BP	0.10 (7)	1.23 (5)	0.43 (6)	0.37 (7)
	CC	0.10 (5)	1.26 (7)	0.50 (3)	0.42 (4)
	MF	0.10 (6)	1.24 (6)	0.42 (8)	0.40 (6)
KEGG		0.10 (8)	1.28 (8)	0.43 (7)	0.31 (8)
MO: Predicted Operons		0.06 (1)	0.92 (1)	0.57 (1)	0.56 (1)
SEED	SS	0.09 (4)	1.19 (4)	0.47 (5)	0.41 (5)
	Scenarios	0.08 (3)	1.06 (3)	0.49 (4)	0.49 (2)
	Paths	0.08 (2)	1.05 (2)	0.50 (2)	0.48 (3)

a. Smaller values mean more consistent sets, because there is less variability within the set.

b. Larger values mean more consistent sets, because the sets contain genes with higher correlations.

### Comparing sources accounting for set size differences

We have already seen that some sources (especially GO and KEGG) can generate extremely large sets. In practice, very large sets are often ignored in gene expression analysis due to both biological and computational practicalities. In the following section we expand our set source comparisons to account for set size differences to ensure that the observed differences in gene set consistency are not attributable to set size differences between the sources.

**Figure 1:**
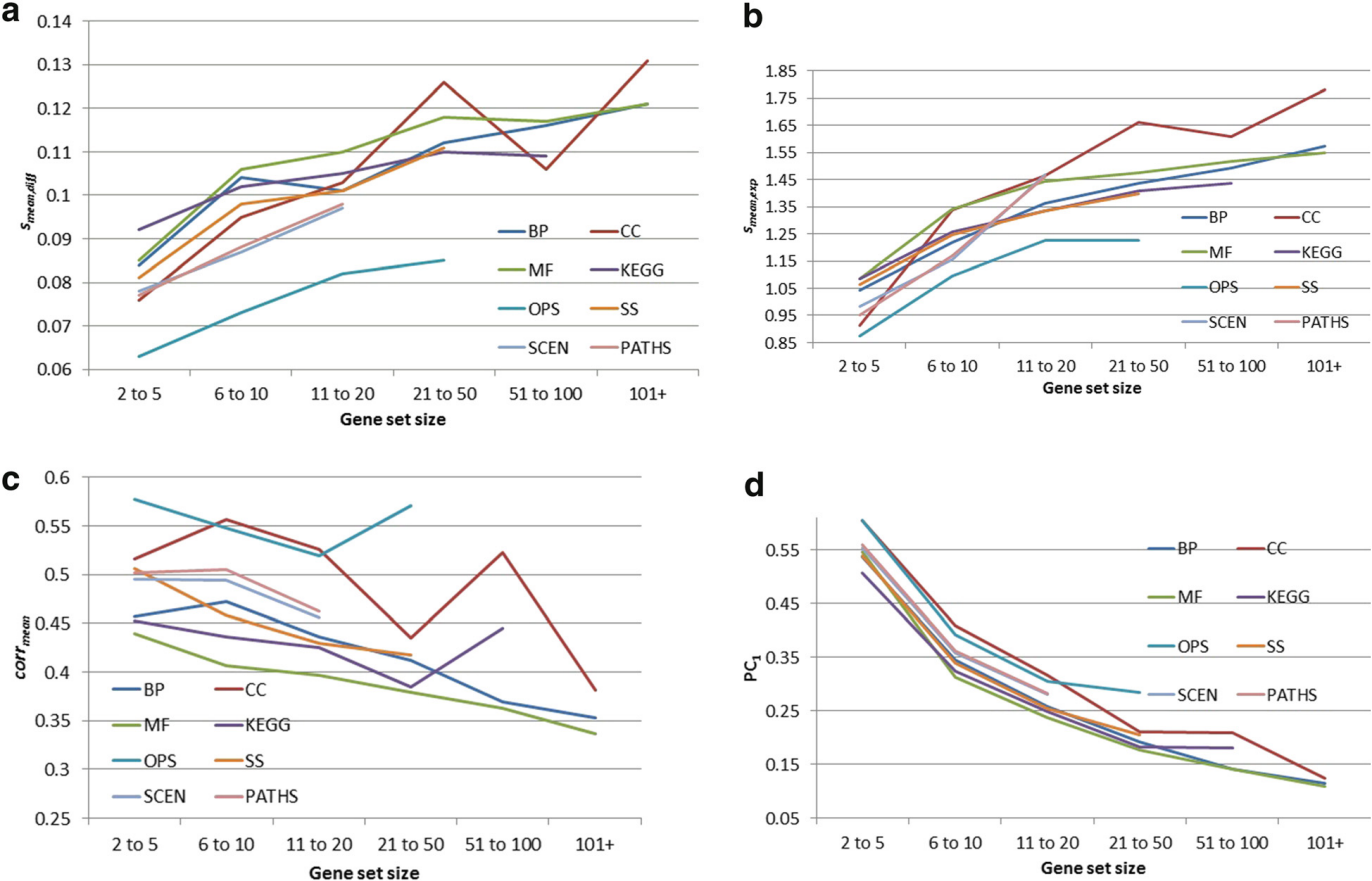
**Gene set consistency by gene set size across the eight gene set sources.a.** Assessing gene set consistency using *s*_*mean,diff*_^*1*^. ^1^ Smaller values of *s*_*mean,exp*_ indicate more consistent sources. **b.** Assessing gene set consistency using *s*_*mean,exp*_^*1*^. ^1^ Smaller values of *s*_*mean,diff*_ indicate more consistent sources. **c.** Assessing gene set consistency using *corr*_*mean*_^*1*^. ^1^Larger values of *corr*_*mean*_ indicate more consistent sources.^1^**d.** Assessing gene set consistency using PC_1_^1^. ^1^ Larger values of PC_1_ indicate more consistent sources

Figures 1a-d show the average value of the four consistency metrics across different set sizes, for each of the eight different sources of sets (we restrict the analysis to combinations of set sources and set sizes containing at least 20 sets). In general, the patterns remain as suggested by Table 5. For all eight sources of sets gene set consistency tends to decrease as set size increases. Furthermore, in general, gene set consistency is impacted similarly for each of the eight sources. Thus, sources of sets that are the best/worst will retain that status across all set sizes. In particular, we note that Predicted Operons were typically the most consistent, followed by Paths and Scenarios, regardless of set size across all four consistency metrics. As observed in Table 5, Cellular Component sets tend to be well correlated, comparable to Predicted Operons, and better than Scenarios/Paths, but perform more poorly than Scenarios/Paths when evaluated by *s*_*mean,diff*_ and *s*_*mean,exp*_ when the Cellular Components sets contain at least 6 genes.

### Comparing sources accounting for set size and organism differences

We recognize that some organisms may, in general, or in particular, provide more or less consistent sets. Thus, we used a general linear modeling approach to evaluate gene set consistency across the eight sources of sets, controlling for both set size and organism. Specifically, we predicted each gene set consistency metric by different combinations of set source, set size (log-transformed number of genes) and organism. The first model only predicted gene set consistency by source, the second model used source and set size, the third used source, set size and organism. The fourth, final model, included source, set size and organism, along with all possible interaction terms between these variables (the first three models only contained the main effects terms).

Detailed results are available in Additional file 1: Table S1 and Additional file 2: Table S2. In general, the relationship of source to gene set consistency remained unchanged in models controlling for set size and organism. In other words, the differences in gene set consistency are not accounted for by set size or organism: The “ranking” of gene set sources based on the four consistency metrics described earlier remains virtually unchanged from Table 4, even after accounting for organism and set size differences. We also found that the most complex linear model (containing all possible interaction terms) explained only modestly more variability in consistency metrics than was explained by the model with only main effects terms. This suggests that while certain organism, set size and source combinations may be particularly good/bad, most of the differences are explained simply by source (e.g., GO poorer than Predicted Operons), set size (larger sets tend to be less consistent) and organism alone (certain organisms tend to have more consistent sets than others). Additional file 3: Table S3 gives the model *r*^*2*^ values (% of total variability in the gene set consistency metric explained by the model) for four different types of models.

### Reduced analyses

SEED Paths have been identified as providing some of the most consistent sets, second only to Predicted Operons. However, Paths are only defined for genes involved in metabolic processes. To evaluate whether the benefits of Paths are due to the way they are constructed, or simply because they cover metabolic processes (and that, perhaps, sets constructed on metabolic processes, regardless of source, will tend to be consistent), we conducted a follow-up analysis similar to Table 5 using only gene sets containing genes present in a Path. In effect, we reduced KEGG, GO, SS and Predicted Operon sets to only those containing metabolic process genes. Additional file 4: Table S4 provides detailed results. In general, the results are similar to those observed in Table 4, with modest improvement for Cellular Component sets. Thus, we find the Cellular Component sets consisting of metabolic process genes tend to perform comparably to Paths/Scenarios across all four consistency metrics. Because Predicted Operons also performed well, we performed the same reduced analysis considering only genes that appear in at least one Predicted Operon. We observed little change in results (see Additional file 5: Table S5).

Furthermore, to ensure that the strong consistency demonstrated by Predicted Operons, Paths and Scenarios is not because these sources have a large degree of overlap in their sets, we computed the percent of genes appearing in more than one set from a source, as a fraction of the total genes appearing at least once in a set for the source. As expected, only 0.1% of the genes in at least one Predicted Operons are in more than one predicted operon. Similarly, only modest overlap in sets was observed for Paths (29.5% of genes in a Path set are in more than one Path set), with similar values for Scenarios (22.6%), KEGG (21.1%) and Subsystems (29.5%) All Gene Ontology based sets exhibited high overlap, with Molecular Function and Biological Process sets showing the largest overlap (60.5% and 75.1% of genes in at least two sets, respectively), while Cellular Component (GO) sets exhibited slightly lower overlap (36.1%.). We also computed, for each combination of source and organism, the proportion of an organism’s genes that were in at least one gene set for that source. Averaging across all organisms we find that 8.5% of genes are in at least one SEED Path, 8.6% are in at least on SEED Scenario, 25.5% are in at least one GO Cellular Component set, 29.9% are in at least one KEGG set, 48.5% are in at least one SEED Subsystem, 49.9% are in at least one GO Biological Process set, 58.2% are in at least one GO Molecular Function set and 79.4% are in at least one MO Predicted Operon. While there were significant differences in these percentages between organisms, the general pattern of results stayed the same.

### Arginine biosynthesis example revisited

Our analysis shows that gene sets derived from Predicted Operons, SEED Paths and Scenarios are the most consistent, while those derived from GO and KEGG tend to be the least consistent. To understand why this may be the case, we further consider the particular example discussed earlier in the Methods (see Table 2).

Table 2 illustrates that SEED Paths and Scenarios provide specific detailed information about the metabolic function of an organism, and provide a specificity of information not captured by related GO sets and KEGG sets. In this example, the GO set does not include genes that are known to be essential to arginine biosynthesis, but for some reason are not annotated with the appropriate GO term. On the other hand, the KEGG sets include numerous other genes that are not directly related to arginine biosynthesis. Lastly, while operons tend to show strong correlation there is little operonal structure in this set of genes.

Additional file 6: Table S6 provides the values of the gene set consistency metrics for each of the sets in Table 2. In short, gene set consistency illustrated in Additional file 6: Table S6 follows the general trends seen earlier. Namely, the GO sets, including the two small sized sets (GO: 0005524 (MF) with 3 genes, and GO: 0006526 (BP) with 9 genes) show less consistency than does the Scenario/Path being illustrated (Glutamate to Arginine (SEED: Scenario/Path) with 9 genes). Additionally, the Scenario/Path (Glutamate to Arginine (SEED: Scenario/Path) eliminates the exact two genes in the Subsystem with the, overall, weakest pairwise correlations with the rest of the members of the set (fig|83333.1.peg.2440 and fig|83333.1.peg.3181; see Additional file 7: Figure 1). Notably, while the one operon shown in Additional file 6: Table S6 has the highest consistency on all metrics, the small drop in gene set consistency from using the scenario/path may, potentially, be worth it due to the increased biological knowledge. Of course, this is dependent upon the goals of the experiments being analyzed, and the true biology of the organism under consideration.

## Discussion

In this manuscript we have provided the first comprehensive, cross-organismal look at bacterial gene expression patterns across multiple gene set sources using a set of gene expression consistency metrics directly related to numerous disparate statistical analysis approaches. Ultimately, we find that MO Predicted Operons perform well across organisms and set sizes, regardless of the analytic approach being used. Scenarios and Paths from the SEED also perform well in a variety of situations. Cellular Component sets from the Gene Ontology perform well in analyses based on correlating pairs of genes. Table 6 summarizes the optimal set sources by statistical method.

**Table 6:** Summary of optimal set sources by statistical method

**Statistical methods**	**Consistency metric**	**Top 3 or 4 most optimal set sources**
Rank ordered differential expression; Gene set analysis on differential expression	*s*_*mean,diff*_	Predicted Operons, Paths, Scenarios
Rank ordered absolute expression; On/off calling algorithms; Flux-balance analysis; Gene set analysis on absolute expression	*s*_*mean,exp*_	Predicted Operons, Paths, Scenarios
Correlation of pairs of genes; K-means clustering; Regulatory Network Inference; Operon prediction	*corr*_*mean*_*, PC*_*1*_	Predicted Operons, Gene Ontology: Cellular Component Hierarchy, Paths, Scenarios

Gene Ontology sets and KEGG sets, which are the most popular choices for statistical analysis of gene expression data, generally perform quite poorly compared to Predicted Operons, Scenarios and Paths. This poor performance is not attributable to differences in set sizes, is consistent across organisms, and is not a result of Predicted Operons/Scenarios/Paths focusing on portions of the genome which provide higher consistency metrics overall.

The source of Paths, Scenarios and Subsystems is the SEED, which was developed on the premise that a key component of improved high-throughput annotation is experts annotating single subsystems over all genomes, rather than all the genes in a single genome [15]. Annotation using the SEED specifically focuses on ensuring that functional subsystems are annotated coherently and completely. The fact that GO may have less rigorous standardized protocols for bacterial genome annotation with GO terms, and thus fails to ensure that functional subsystems are annotated coherently and completely may be contributing to less consistency in bacterial expression data for GO based sets [24,25].

As illustrated with the example of arginine biosynthesis, GO and KEGG tend to link together disparate sets of genes, translating into lower consistency metrics across organisms. The widespread use of less consistent sets is important because it translates directly into low statistical power when conducting gene expression data analysis, meaning that the application of GO and KEGG gene sets to gene expression data is significantly weakening the ability to make global biological conclusions from gene expression data. Thus, use of Predicted Operons, Scenarios, Paths and the GO: Cellular Component hierarchy when analyzing bacterial gene expression data should improve statistical power across numerous statistical analysis techniques, and, ultimately, yield increased and improved biological conclusions.

However, there are limitations of Predicted Operons, Scenarios, Paths and the GO: Cellular Component hierarchy. First, while operons are important in some analyses (e.g., regulatory network inference), genes in the same operon are not necessarily of the same function, and so may have limited immediate biological meaning. This limitation of operons is an inherent advantage of Scenarios and Paths which are directly related to metabolic function. So, while Predicted Operons may tend to perform better than Scenarios/Paths overall, the biological utility of Scenarios and Paths may be greater. However, we note that Scenarios and Paths are computed only on central and intermediary metabolism, and so are limited in their utility for research questions outside of that area. Additionally, the GO: Cellular component hierarchy, performed well on correlation metrics and so may have utility for statistical analysis approaches based on correlation of expression profiles (e.g., regulatory network inference). However, like Scenarios and Paths, the CC hierarchy covers a limited number of genes.

We note that our use of the GO, KEGG, SEED and Microbes Online was designed to represent a typical approach to utilizing the most common resources. More sophisticated approaches (e.g., leveraging network topology in the analysis, use of evidence codes) and alternative databases may yield different results. In our analysis we did not explicitly model inter-set separation, though, as shown, GO sets showed significantly more overlap. While our analyses are based on standard statistical approaches to integrating gene set information which typically do not consider inter-set separation, further work could consider the impact of inter-set separation on our findings. Furthermore, while our analysis focuses on statistical power, use of Paths, Scenarios and Predicted Operons in the analysis of real data is needed to validate that improved statistical power, ultimately, yields improved biological conclusions. Lastly, we note that numerous approaches to evaluating semantic similarity between gene annotations have been proposed to assess the quality of ontologies [e.g., [25,26]. Our approach is fundamentally different than these in that our focus is on evaluating the statistical power of methods that use ontologies as a source of gene sets. Further work is needed to better understand qualitative differences in the semantic similarity approach and our approach.

It is also important to note that our conclusions about GO and KEGG are limited to bacteria only. Furthermore, our conclusions about the utility of GO and KEGG only apply to the statistical analysis of expression data using methods described earlier. In sum, we have demonstrated that for a diverse set of bacteria, Predicted Operons, Scenarios, Paths and the GO: Cellular Component provide more consistently expressed sets of genes, which translate into improved statistical power.

## Conclusions

Ultimately, our analysis argues strongly for the increased use of MicrobesOnline Predicted Operons, SEED-based Scenarios/Paths and the Gene Ontology Cellular component hierarchy in the analysis of bacterial gene expression data across a variety of widely used statistical analysis approaches. While our results suggest that increased statistical power will be obtained through the use of MicrobesOnline Predicted Operons, SEED-based Scenarios/Paths and the Gene Ontology Cellular component sets, further analysis is needed to ensure statistical improvements translate to improved biological interpretation. Additionally, comparative analyses are needed to explore increased use of SEED-based Scenarios/Paths and MicrobesOnline Predicted Operons for analyses that do not involve gene expression data.

## Competing interests

The authors declare that they have no competing interests.

## Authors’ contributions

NT, AB and MDJ conceived of the study, supervised the analysis and drafted the manuscript. AS, BB and KY conducted the analysis and drafted early versions of the manuscript. All authors read and approved the final manuscript.

## Supplementary Material

Additional file 1 **Table S1. **Marginal effects of source in models controlling for set size only. Click here for file

Additional file 2 **Table S2. **Marginal effects of source in models controlling for set size and organism. Click here for file

Additional file 3 **Table S3. **Model r^2^ when controlling for set size and organism. Click here for file

Additional file 4 **Table S4. **Mean levels of consistency by source when sets are reduced to only contain genes that are contained in at least one SEED path. Click here for file

Additional file 5 **Table S5. **Mean levels of consistency by source when sets are reduced to only contain genes that are contained in at least one predicted operon. Click here for file

Additional file 6 **Table S6. **Gene set consistency characteristics for sets associated with arginine biosynthesis. Click here for file

Additional file 7 **Figure S1.** Pairwise correlations between genes in arginine biosynthesis. Click here for file
