# Supplementary material for: Evaluating the consistency of gene sets used in the analysis of bacterial gene expression data
Source: BMC Bioinformatics. 2012 Aug 8;13:193. doi: 10.1186/1471-2105-13-193 (PMC3462729; doi:10.1186/1471-2105-13-193)
Supplement: Additional file 1 — Table S1. Marginal effects of source in models controlling for set size only. [file 1471-2105-13-193-S1.pdf]

Supplemental Table 1. Marginal effects[1] of source in models controlling for set size only

		$s_{mean,diff}$	$s_{mean,exp}$	$corr_{mean}$	PC_1
Gene Ontology	BP	0.022	0.15	-0.10	-0.06
	CC	0.019	0.17	-0.04	0.01
	MF	0.025	0.21	-0.14	-0.06
KEGG		0.025	0.17	-0.11	-0.09
MO Predicted Operons[2]		0	0	0	0
SEED	SS	0.020	0.18	-0.08	-0.08
	Scenarios	0.015	0.11	-0.07	-0.05
	Paths	0.014	0.09	-0.07	-0.05

1. Marginal effects are interpreted as the average difference in consistency metric comparing the source to predicted operons. For example, sets from the Gene Ontology BP, on average and controlling for set size differences, have values of $s_{mean,diff}$ which are 0.022 larger (lower consistency) than predicted operons. Note that positive marginal effects for $s_{mean,diff}$ and $s_{mean,exp}$, and negative marginal effects for $corr_{mean}$ and PC_1 all indicate that, on average, the source provides lower consistency than predicted operons.
2. Predicted operons were the reference category in the model and so marginal effects are interpreted as the average difference in the consistency metric for the source vs. operons
