# Supplementary material for: Evaluating the consistency of gene sets used in the analysis of bacterial gene expression data
Source: BMC Bioinformatics. 2012 Aug 8;13:193. doi: 10.1186/1471-2105-13-193 (PMC3462729; doi:10.1186/1471-2105-13-193)
Supplement: Additional file 3 — Table S3. Model r2 when controlling for set size and organism. [file 1471-2105-13-193-S3.pdf]

Supplemental Table 3. Model r^2 when controlling for set size and organism

Consistency Metric	Source only	Source+Set size	Source+Set size+Organism	Source*SetSize*Organism
$s_{mean,diff}$	6.8%	11.4%	51.1%	55.1%
$s_{mean,exp}$	5.6%	14.0%	20.1%	23.6%
$corr_{mean}$	6.6%	8.0%	29.7%	35.7%
PC_1	15.0%	64.5%	71.7%	75.8%
