# Supplementary material for: Evaluating the consistency of gene sets used in the analysis of bacterial gene expression data
Source: BMC Bioinformatics. 2012 Aug 8;13:193. doi: 10.1186/1471-2105-13-193 (PMC3462729; doi:10.1186/1471-2105-13-193)
Supplement: Additional file 4 — Table S4. Mean levels of consistency by source when sets are reduced to only contain genes that are contained in at least one SEED path. [file 1471-2105-13-193-S4.pdf]

Supplemental Table 4. Mean levels of consistency by source (rank out of the 8 sources in parentheses) when sets are reduced to only contain genes that are contained in at least one SEED path

		$s_{mean,diff}$	$s_{mean,exp}$	$corr_{mean}$	PC_1
Gene Ontology	BP	0.09 (5)	1.10 (5)	0.47 (5)	0.42 (7)
	CC	0.09 (4)	1.09 (4)	0.54 (2)	0.51 (2)
	MF	0.10 (8)	1.18 (7)	0.43 (8)	0.44 (6)
KEGG		0.10 (7)	1.19 (8)	0.45 (7)	0.38 (8)
MO Predicted Operons		0.05 (1)	0.67 (1)	0.71 (1)	0.68 (1)
SEED	SS	0.09 (6)	1.15 (6)	0.46 (6)	0.44 (5)
	Scenarios	0.08 (3)	1.04 (2)	0.50 (4)	0.49 (3)
	Paths	0.08 (2)	1.05 (3)	0.50 (3)	0.48 (4)
