# Supplementary material for: Evaluating the consistency of gene sets used in the analysis of bacterial gene expression data
Source: BMC Bioinformatics. 2012 Aug 8;13:193. doi: 10.1186/1471-2105-13-193 (PMC3462729; doi:10.1186/1471-2105-13-193)
Supplement: Additional file 6 — Table S6. Gene set consistency characteristics for sets associated with arginine biosynthesis. [file 1471-2105-13-193-S6.pdf]

Supplemental Table 6. Gene set consistency characteristics[1] for sets associated with arginine biosynthesis

	Number of genes	$s_{mean,exp}$	$s_{mean,diff}$	$corr_{mean}$	PC_1
GO: 0006526 (BP)	9	0.08	0.72	0.45	0.32
GO: 0004055 (MF)	336	0.09	1.17	0.24	0.05
GO: 0005524 (MF)	3	0.08	0.89	0.19	0.40
Alanine, aspartate and glutamate metabolism (eco00250:KEGG)	29	0.18	1.29	0.28	0.13
Arginine and proline metabolism (eco00330:KEGG)	43	0.10	1.01	0.26	0.10
Arginine Biosynthesis extended (SEED:SS)	11	0.07	0.55	0.56	0.34
Glutamate to Arginine (SEED: Scenario/Path)	9	0.06	0.51	0.78	0.44
MO Predicted Operon (peg.3878,3879,3880)	3	0.05	0.40	0.87	0.71

[1] Smaller values of $s_{mean,exp}$ and $s_{mean,diff}$ indicate more consistent sources. While, larger values of $corr_{mean}$ and PC_1 indicate more consistent sources.
