# Supplementary figures and images for: Evaluating the consistency of gene sets used in the analysis of bacterial gene expression data
Source: BMC Bioinformatics. 2012 Aug 8;13:193. doi: 10.1186/1471-2105-13-193 (PMC3462729; doi:10.1186/1471-2105-13-193)

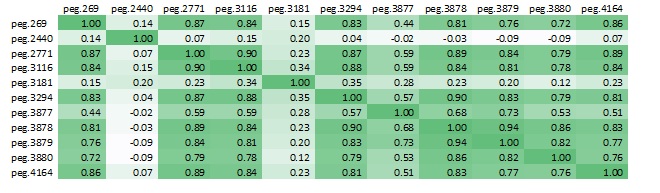

Supplement: Additional file 7 — Figure S1. Pairwise correlations between genes in arginine biosynthesis. [file 1471-2105-13-193-S7.jpeg]
